# Interpreting the MicroRNA-15/107 family: interaction identification by combining network based and experiment supported approach

**DOI:** 10.1186/s12881-019-0824-9

**Published:** 2019-05-31

**Authors:** Si Wang, Wenhua Zhu, Jing Xu, Yuanxu Guo, Jidong Yan, Liesu Meng, Congshan Jiang, Shemin Lu

**Affiliations:** 1 Department of Biochemistry and Molecular Biology, School of Basic Medical Sciences, Xi’an Jiaotong University Health Science Center, Xi’an, Shaanxi 710061 People’s Republic of China; 2 Key Laboratory of Environment and Genes Related to Diseases (Xi’an Jiaotong University), Ministry of Education, Xi’an, Shaanxi 710061 People’s Republic of China; 3 Department of Human Anatomy, Histology and Embryology, School of Basic Medical Sciences, Xi’an Jiaotong University Health Science Center, Xi’an, Shaanxi 710061 People’s Republic of China

**Keywords:** miR-15/107 family, Target gene, Bioinformatics, Interaction network

## Abstract

**Background:**

The highly conservative miR-15/107 family (also named as miR-15/107 gene group) including ten miRNA members is currently recognized strongly implicated in multiple human disorders. Some studies focus on the entire family rather than individual miRNA for a bigger picture, while there is also certain signature dysregulation for some of the individual miRNA implicated even in the same disorder.

**Methods:**

Faced with the exponential growth of experimental evidence, our study tries to analyze their function and target interactions using various bioinformatics tools.

**Results:**

Firstly, the evolutionary conservative “AGCAGC” sequence and possible clustered transcriptional pattern were described. Secondly, both the experimentally validated and bioinformatically predicted miRNA-target gene relationship of the entire family was analyzed to understand the mechanism of underlying collective effects for target regulation from the miR-15/107 family. Moreover, pathway analysis among miR-15/107 family was performed and displayed in detail, while its impact on cell proliferation is experimentally validated. Eventually, the dysregulation of miR-15/107 in diseases was discussed.

**Conclusions:**

In summary, our study proposes that the collective functions and implication of miR-15/107 family in various human diseases are achieved relying on the massive overlapping target genes. While the minor differences within target gene interaction among family members could also explain the signature behavior for some of the individual miRNA in aspects such as its disease-specific dysregulation and various participation in pathways.

**Electronic supplementary material:**

The online version of this article (10.1186/s12881-019-0824-9) contains supplementary material, which is available to authorized users.

## Background

### General background of evolutionarily conservative miR-15/107 family

The miR-15/107 family (also called as miR-15/107 gene group) contains multiple highly conservative miRNA members, including miR-15a-5p, miR-15b-5p, miR-16-5p, miR-103a-3p, miR-107, miR-195-5p, miR-424-5p, miR-497-5p, miR-503-5p and miR-646 [1]. Mature miRNA form of this entire family was found highly expressed in eleven human tissues including the cerebral cortex, frontal cortex, primary visual cortex, thalamus, heart, lung, liver, kidney, spleen, stomach and skeletal muscle [2]. These miRNAs share a common “AGCAGC” sequence within the crucial No.2-No.7 seed binding region for their targets [1], which leads to the potential functional overlap among this family.

### Family behavior vs. individual signature

Since this miRNA family was recognized [3], the researchers started to capture the impact of the entire family rather than focus exclusively on a single one of such miRNA, which helped us better understand their biological role [4]. The entire miR-15/107 family was found to suppress the BRCA1 gene at a post-transcriptional level in nine cell lines [5]. The mitogen and growth factor Granulin was dysregulated resulted from the miR-15/107 gene group in multiple human cancers [6]. The miR-15/107 family was found to regulate its target gene CDK5R1/p35 during Alzheimer’s disease (AD) pathogenesis [7].

Besides the important family behavior of miR-15/107, there was also certain signature expression profile for some of individual miRNA implicated even in the same disorder. For example, multiple miR-15/107 family members were developed for therapy of Alzheimer’s disease (AD), among which the miR-16 was selected as the best candidate for simultaneously suppressing multiple AD biomarkers including Aβ and Tau [8]. The investigation focused on this same disorder discovered that all the other members of the miR-15/107 family were down-regulated in gray matter of temporal cortex from the AD patients except for the miR-424, which was up-regulated in white matter in AD [9].

### Aim of study

In short, the miR-15/107 family is widely considered with strong influences on human biology in health and disease. Faced with the exponential growth of experimental evidence as well as the mounting bioinformatics tools for miRNA dissection [10], this study aims to analyze the target interactions and its implication in multiple pathways and various diseases.

## Methods

### miRNA sequence alignment

Analysis of “AGCAGC” sequence within mature miRNA molecules was achieved according to the “mature.fa” dataset downloaded from miRBase website, a well-recognized online repository of miRNA sequences and associated annotation [11–15].

### Search for genomic position of miRNA host genes

miRNA host genes were found with help of the UCSC Genome Browser [16], a graphical viewing and analyzing tool for genomic information based on the human genome assemblies and annotations.

### miRNA-mRNA target relationship analysis

miRNA-mRNA target was analyzed for both experimentally validated interactions using TarBase v8.0 and computationally predicted interactions using Targetscan database (release 7.2, updated in March 2018) [17].

### Analysis of multi-miRNA regulatory network

miRTargetLink database calculates the integrated multi-miRNA mode of regulatory network for the understanding of collective effects according to the experimentally validated targets with strong or weak evidence [18].

### miRNA pathway analysis

The miRNA regulated KEGG pathway [19] was analyzed using DIANA-miRPath v3.0 [20], a web-server using merging and meta-analysis algorithms according to either predicted or experimentally validated miRNA target interactions. Here in this study, we chose to analyze the validated miRNA interactions using archive in TarBase [21]. Using this algorithm, *P* < 0.05 in Fisher’s Exact Test (for hypergeometric distribution) was considered as statistically significant.

### Cell proliferation assay

SW982 cells were cultured in DMEM high glucose medium (Hyclone, USA) supplemented with 10% FBS (Hyclone, USA) and 0.2% penicillin/streptomycin. Gain of miR-15/107 function was achieved by transfecting 10 nM miRNA mimic (Genepharma company, China) with Lipofectamine 3000 reagent (Invitrogen, USA) according to the manufacturer’s instruction. The scramble miRNA mimic served as negative control (NC). 48 h after cell transfection, CCK8 reagent was applied to the cells and incubated at 37 °C for 1 h. Optical density value was measured at 450 nm. The data was further normalized as percentage against NC group. Results for cell proliferation assay are represented as the mean ± SEM from the three independent cell experiments. Mann-Whitney test was used to analyze the statistical difference between the indicated group and NC group. *P* value less than 0.05 was considered as statistically significant.

### Analysis of dysregulated miR-15/107 in disease

miR2Disease is a manually curated database offering the miRNA-disease relationship, miRNA expression pattern in the disease state, the miRNA expression detection method, experimentally verified miRNA target genes and literature references [22]. With the help of miR2Disease, we could understand the comprehensive potential role in disease state.

## Results

### The “AGCAGC” sequence for evolutionarily conservative miR-15/107 family

According to the archive in miRBase (Release 22, updated in March 2018), the miR-15/107 family (such as miR-15a-5p, miR-15b-5p, miR-16-5p, miR-103a-3p, miR-107, miR-195-5p, miR-424-5p, miR-497-5p, miR-503-5p and miR-646) all shared an evolutionarily conservative “AGCAGC” sequence at the 5′ end of the miRNAs (Table 1). After investigating all the 2656 mature human miRNAs identified up to date, we found that another miRNA named miR-6838-5p also contains the specific “AGCAGC” sequence at the seed binding region. The systemic concept of miR-15/107 family was characterized in 2010 [1], before the discovery of miR-6838-5p using RNA sequencing in mice and human [23]. Hence, in this study we also included it for further analysis. In addition, there are five other miRNAs (miR-191-5p, miR-4640-5p, miR-6762-5p, miR-6812-5p and miR-6868-5p) harboring an “AGCAGC” sequence within its full length, while for now there is no evidence for any similarity in the potential target binding function with their targets.

**Table 1:** The “AGCAGC” sequence for evolutionarily conservative miR-15/107 family

miRNAs	miRBase Accession Number	Sequence (from 5′ to 3′)
hsa-miR-15a-5p	MIMAT0000068	UAGCAGCACAUAAUGGUUUGUG
hsa-miR-15b-5p	MIMAT0000417	UAGCAGCACAUCAUGGUUUACA
hsa-miR-16-5p	MIMAT0000069	UAGCAGCACGUAAAUAUUGGCG
hsa-miR-103a-3p	MIMAT0000101	AGCAGCAUUGUACAGGGCUAUGA
hsa-miR-107	MIMAT0000104	AGCAGCAUUGUACAGGGCUAUCA
hsa-miR-195-5p	MIMAT0000461	UAGCAGCACAGAAAUAUUGGC
hsa-miR-424-5p	MIMAT0001341	CAGCAGCAAUUCAUGUUUUGAA
hsa-miR-497-5p	MIMAT0002820	CAGCAGCACACUGUGGUUUGU
hsa-miR-503-5p	MIMAT0002874	UAGCAGCGGGAACAGUUCUGCAG
hsa-miR-646	MIMAT0003316	AAGCAGCUGCCUCUGAGGC
hsa-miR-6838-5p	MIMAT0027578	AAGCAGCAGUGGCAAGACUCCU

### Possible transcriptional pattern in clusters

To figure out the possible underlying mechanism of the dysregulated miRNA expression profiles in health and disease at transcriptional level, the precursor transcripts of these miRNAs were searched for gene clusters (host gene and chromosome position of these miRNAs were detailed in Table 2). With the help of UCSC Genome Browser, it was found that there were 4 pairs of neighboring miRNA clusters including miR-15a-5p/miR-16-5p, miR-15b-5p/miR-16-5p, miR-195-5p/ miR-497-5p, miR-424-5p/miR-503-5p within the common host genes. Moreover, the host genes of miR-103a-3p and miR-107 independently but coincidently belong to the pantothenate kinase family, even though located at entirely different chromosomes. All the above-mentioned clues indicated that the miR-15/107 family might be synergistically transcribed in pairs except for the miR-646 and miR-6838-5p.

**Table 2:** The precursor transcripts of the miR-15/107 family

miRNAs	Host gene	Transcript location	Chromosome position
hsa-miR-15a-5p	deleted in lymphocytic leukemia 2 (DLEU2) non-protein coding gene	Intron	chr13: 50049119–50,049,201 [−]
hsa-miR-15b-5p	structural maintenance of chromosomes 4 (SMC4) protein-coding gene	Intron	chr3: 160404588–160,404,685 [+]
hsa-miR-16-5p	structural maintenance of chromosomes 4 (SMC4) protein-coding gene	Intron	chr3: 160404745–160,404,825 [+]
	deleted in lymphocytic leukemia 2 (DLEU2) non-protein coding gene	Intron	chr13: 50048973–50,049,061 [−]
hsa-miR-103a-3p	pantothenate kinase 3 (PANK3) protein-coding gene	Intron	chr5: 168560896–168,560,973 [−]
	pantothenate kinase 2 (PANK2) protein-coding gene	Intron	chr20: 3917494–3,917,571 [+]
hsa-miR-107	pantothenate kinase 1 (PANK1) protein-coding gene	Intron	chr10: 89592747–89,592,827 [−]
hsa-miR-195-5p	mir-497-195 cluster host gene (MIR497HG) long non-coding RNA	N/A	chr17: 7017615–7,017,701 [−]
hsa-miR-424-5p	MIR503 host gene (MIR503HG) long non-coding RNA	N/A	chrX: 134546614–134,546,711 [−]
hsa-miR-497-5p	mir-497-195 cluster host gene (MIR497HG) long non-coding RNA	N/A	chr17: 7017911–7,018,022 [−]
hsa-miR-503-5p	MIR503 host gene (MIR503HG) long non-coding RNA	N/A	chrX: 134546328–134,546,398 [−]
hsa-miR-646	MIR646 host gene (MIR646HG) long non-coding RNA	N/A	chr20: 60308474–60,308,567 [+]
hsa-miR-6838-5p	polymerase (DNA) mu (POLM) protein-coding gene	Exon of transcript variant 2, intron of transcript variant 1 and 3	chr7: 44073378–44,073,433 [−]

*N/A* not applicable

### Interaction of miR-15/107 family and their target genes

**Fig. 1:**
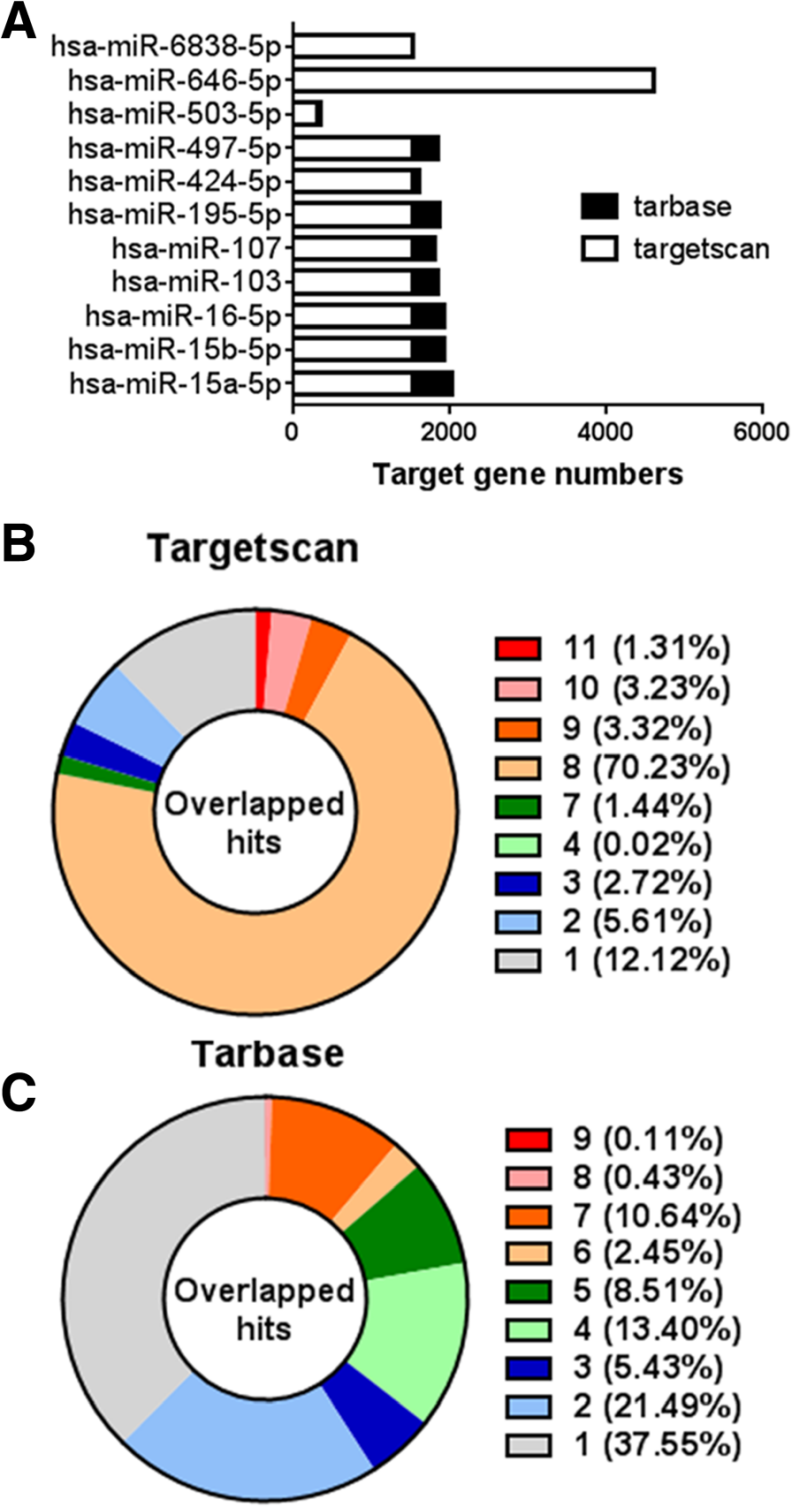
Intersection of miR-15/107 regulated target genes. **a**. Numbers of computationally predicted (archived in Targetscan database) and experimentally validated (archived in TarBase database) target genes. **b**. Computationally predicted target genes regulated by multiple miR-15/107 members. **c**. Experimentally validated target genes regulated by multiple miR-15/107 members

Mounting evidence and bioinformatics tools for miRNA dissection provided us lots of information for miR-15/107 regulated target genes (Fig. 1a). As above-mentioned, the common “AGCAGC” sequence within the seed binding region is the crucial characteristic of miR-15/107 family. Hence, plenty of shared target genes could be widely expected. There are plenty of attributes shared in common within the miR-15/107 family. However, there are also quite a lot of differences in the target gene profiles among individual members which defined their various function (Fig. 1b and c). The similarity and difference of both the computationally predicted and experimentally validated target gene profiles were analyzed and displayed in Additional files 1 and 2. The results showed that most of these target genes (above 78%) were predicted to be overlapping for more than 8 miR-15/107 members, while the experimentally validated miRNA-target interaction archived in TarBase was relatively less informative for those types of target genes. It was possible that either plenty of predicted targets might be false positive, or the present attention was not paid enough for the part of target genes. So far, the numbers for miR-424-5p, miR-503-5p targets were relatively less than the others, while the miRNA targets for miR-646-5p and miR-6838-5p was still absent in TarBase database.

**Fig. 2:**
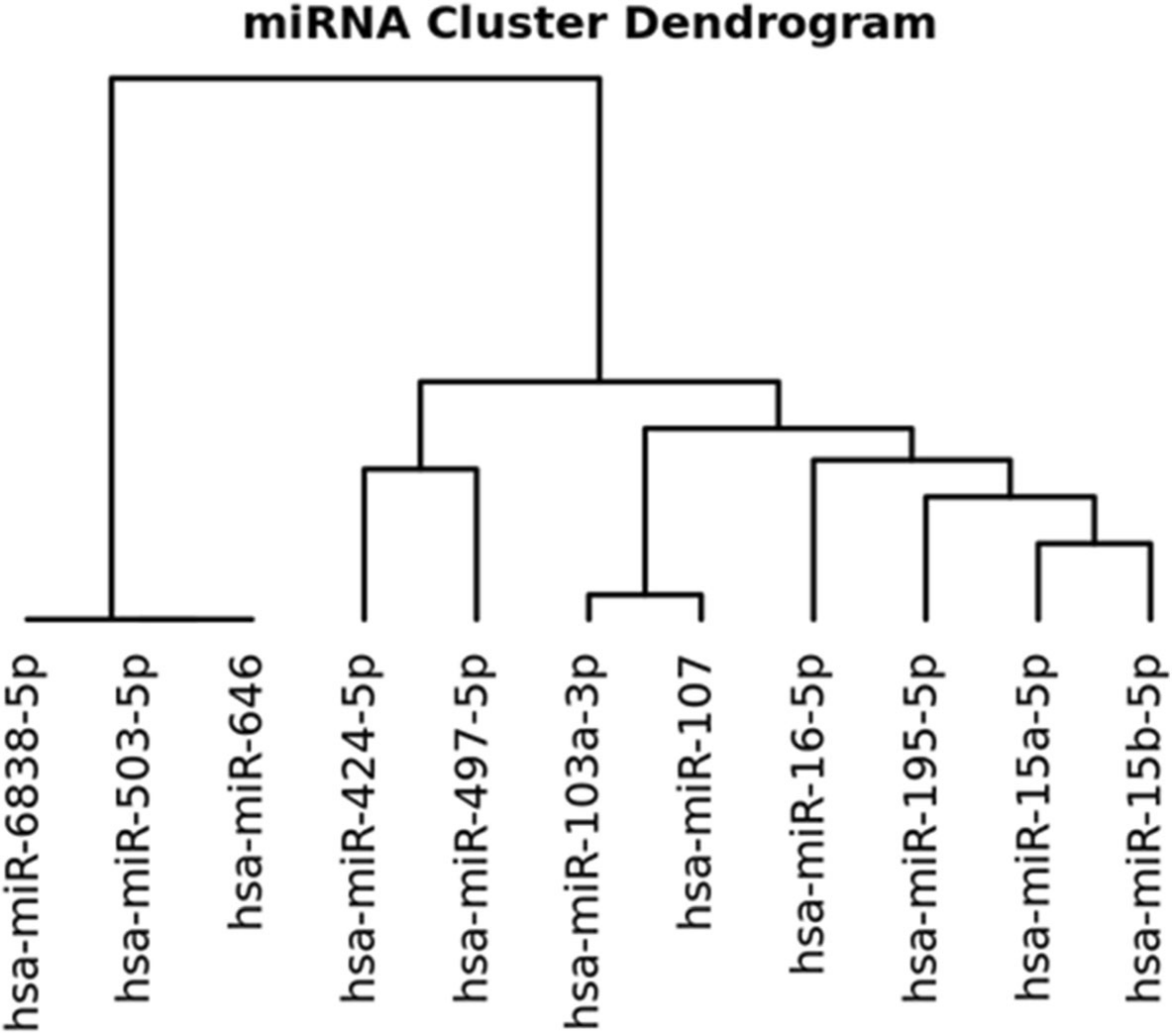
Cluster dendrogram of miR-15/107 family according to the overlapping miRNA-target gene relationship archived in TarBase (a database for experimentally validated miRNA-target gene recognition)

The overlapping target genes were analyzed and a cluster dendrogram of miR-15/107 family was plotted accordingly (Fig. 2). Concerning the profile of their target genes, it was shown that there are three pairs of miRNAs including miR-15a-5p/miR-15b-5p, miR-103a-3p/miR-107 and miR-424/miR-497 closely resembling each other, while three of them including the miR-6838-5p, miR-503-5p and miR-646 vary independently.

### Collective effects of target regulation from the miR-15/107 family

**Fig. 3:**
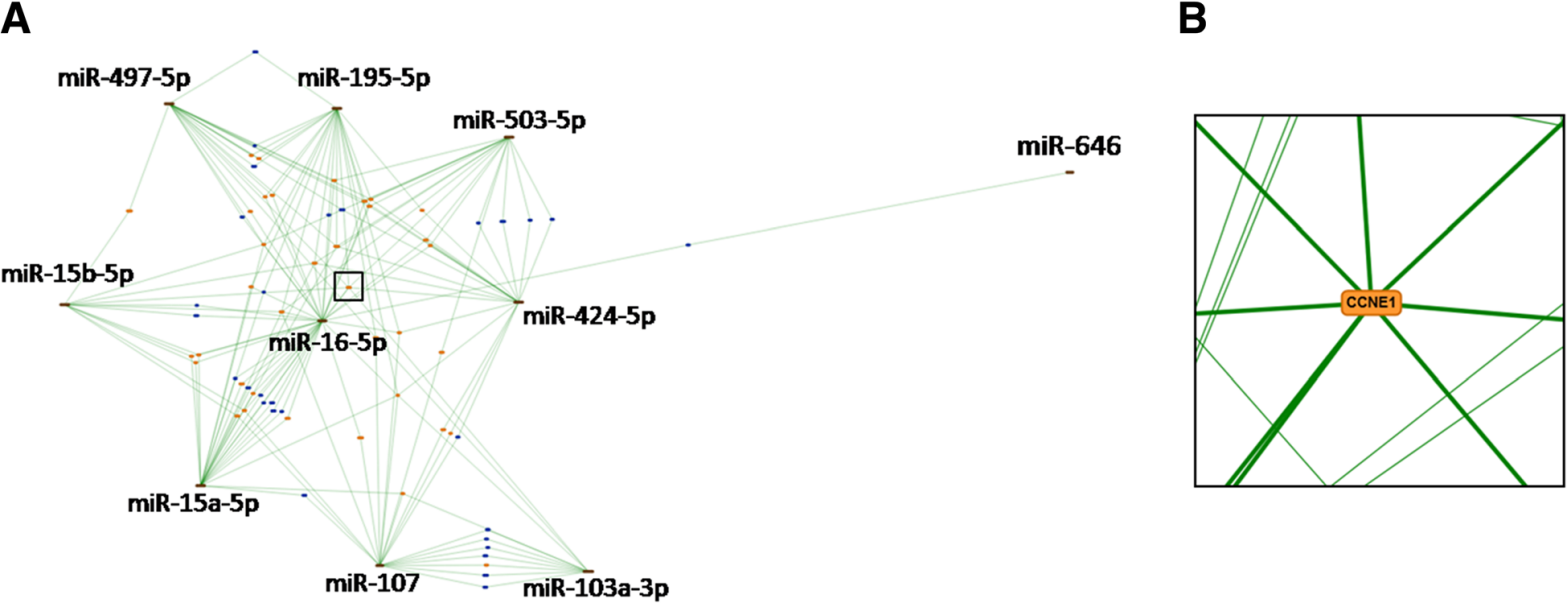
The multi-miRNA and target gene interaction network for miR-15/107 family. **a**. The general outline of miR-15/107 interaction network calculated by miRTargetLink database based on target evidence. miRNA displayed as the edges, and target genes as the nodes of the network. Blue color refers to target genes with 2 interactions, while orange color refers to target genes with more than 2 interactions. **b**. Indicated central part from A was enlarged for details. Green bold lines indicate the target interactions of CCNE1 (node) from 8 various miRNAs (edge)

Since there are massive overlapping target genes among the miR-15/107 family, we further calculated and emphasized the collective effects of target regulation from multi members. According to the regulatory network calculated by miRTargetLink database, we can see that dozens of target genes (located in the central part of this network) could be affected by this family collectively, especially for genes such as CCNE1, CCND1, VEGFA and so on which were simultaneously subject to regulation from more than 5 members of this family (Fig. 3).

### Pathway analysis of the miR-15/107 family

**Fig. 4:**
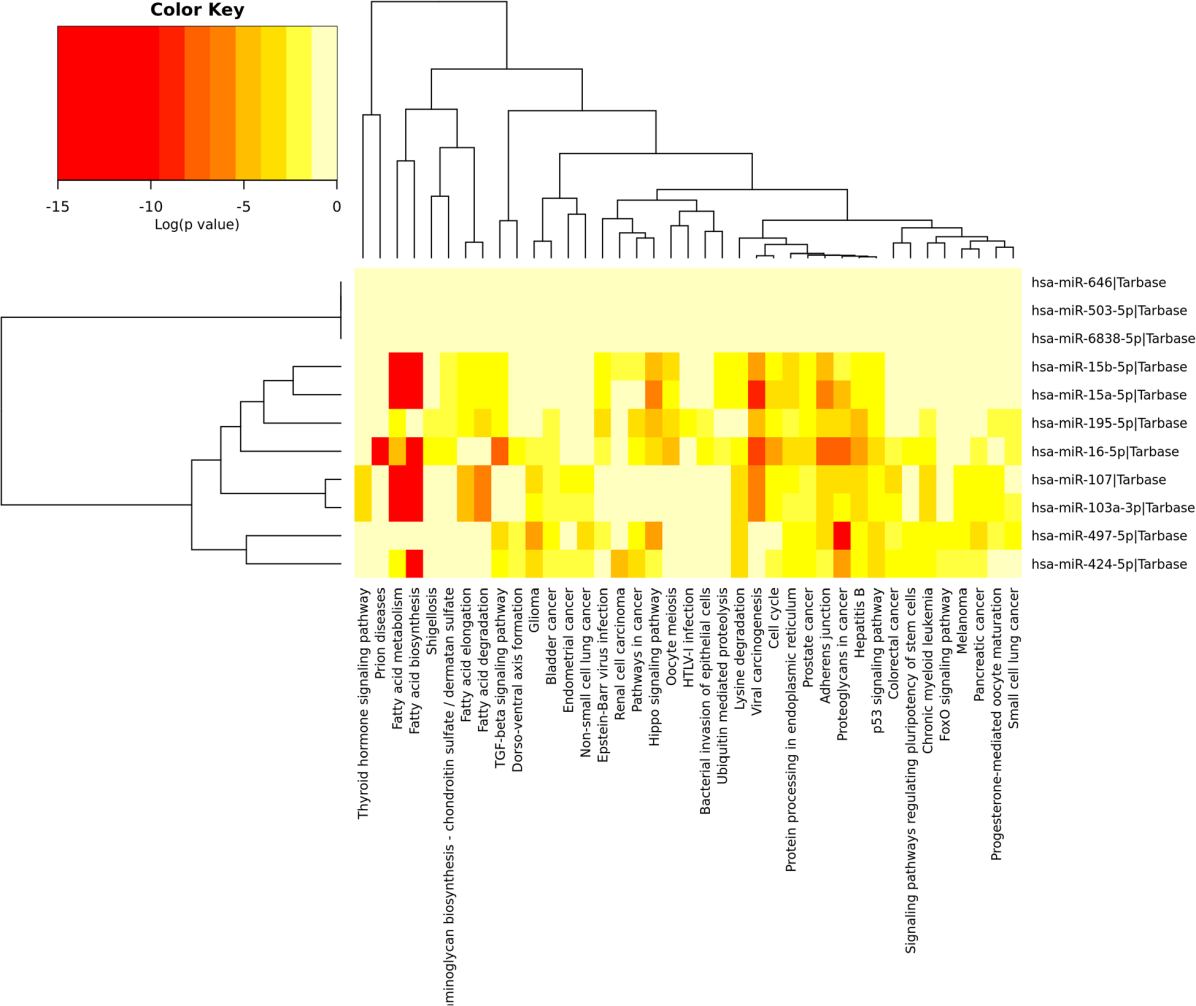
Cluster dendrogram of miR-15/107 significantly regulated KEGG pathways calculated by miRPath v3.0 web-server

miR-15/107 regulated pathways were calculated using mirPath v3.0 web-server, and the results showed that 39 KEGG pathways (see Additional file 3) were significantly (*P* < 0.05 in Fisher’s Exact Test) regulated by the miR-15/107 family (Fig. 4). Pathway calculation was based on merging and meta-analysis algorithms according to experimentally validated miRNA target interactions, hence we could find that cluster dendrogram based on miRNA regulated pathways was consistent with that of miRNA target gene. The pathways regulated by the miR-15a-5p/miR-15b-5p, miR-103a-3p/miR-107 and miR-424/miR-497 closely resembled each other, while the miR-6838-5p, miR-503-5p and miR-646 vary independently. Besides, the cluster dendrogram of these pathways further showed that the most significantly regulated pathways included the fatty acid metabolism/biosynthesis/degradation/elongation, various signaling during carcinogenesis, and some crucial pathways for cell survival such as cell cycle, meiosis, adherent junction et al.

### Cell cycle as a representative pathway regulated by the miR-15/107 family

**Fig. 5:**
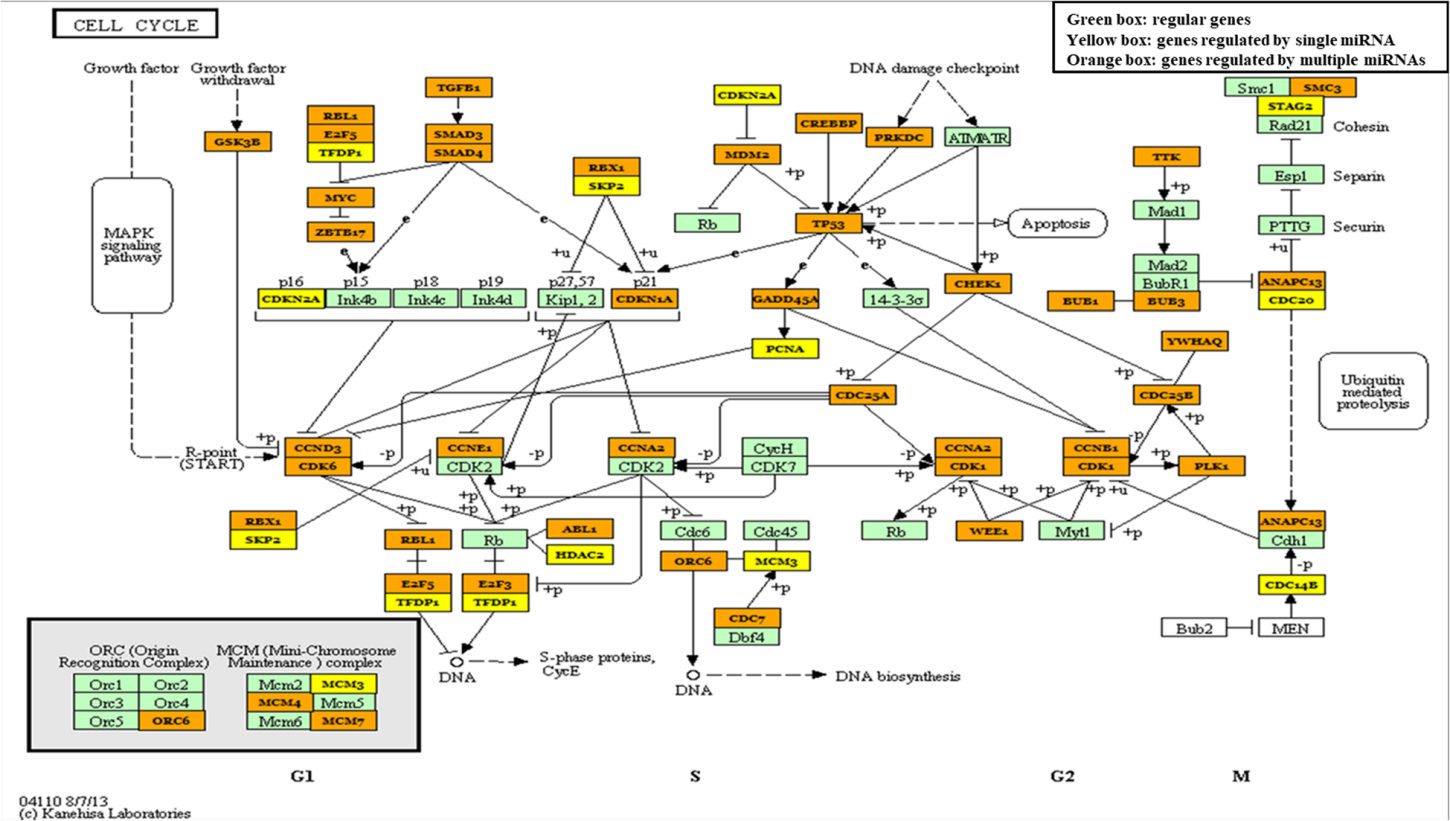
miR-15/107 family targeted genes in cell cycle KEGG pathway calculated by miRPath v3.0 web-server. Green box: regular genes. Yellow box: genes regulated by single miRNA. Orange box: genes regulated by multiple miRNAs

Here, we take the cell cycle pathway as an example, to uncover the implication of miR-15/107 family in determining cell fate. miR-15/107 targeted genes within the cell cycle pathway was highlighted in Fig. 5 and listed in Additional file 4. The results showed that 47 of 65 cell cycle related genes (up to 72%) were regulated by miR-15/107 family. Particularly, some of these target genes play multi-functional roles as molecular contacts within the pathway, such as CDKN2A, RBL1, E2F5, TFDP1, RBX1, SKP2, and MCM3. Besides, cell cycle related complex machineries including ORC (origin recognition complex) and MCM (mini-chromosome maintenance complex) were also regulated by miR-15/107. More importantly, there were up to 15 genes subject to regulation from more than 5 members from the miR-15/107 family simultaneously. These bioinformatics evidence suggested a strong implication of miR-15/107 family in cell cycle regulation, while such prediction was also widely supported by experimental evidence (see Additional file 5). Taken together, miR-15/107 family was strongly implicated in multiple pathways including the cell cycle regulation.

### Members of the miR-15/107 gene family have an inhibitory effect on cell proliferation

**Fig. 6:**
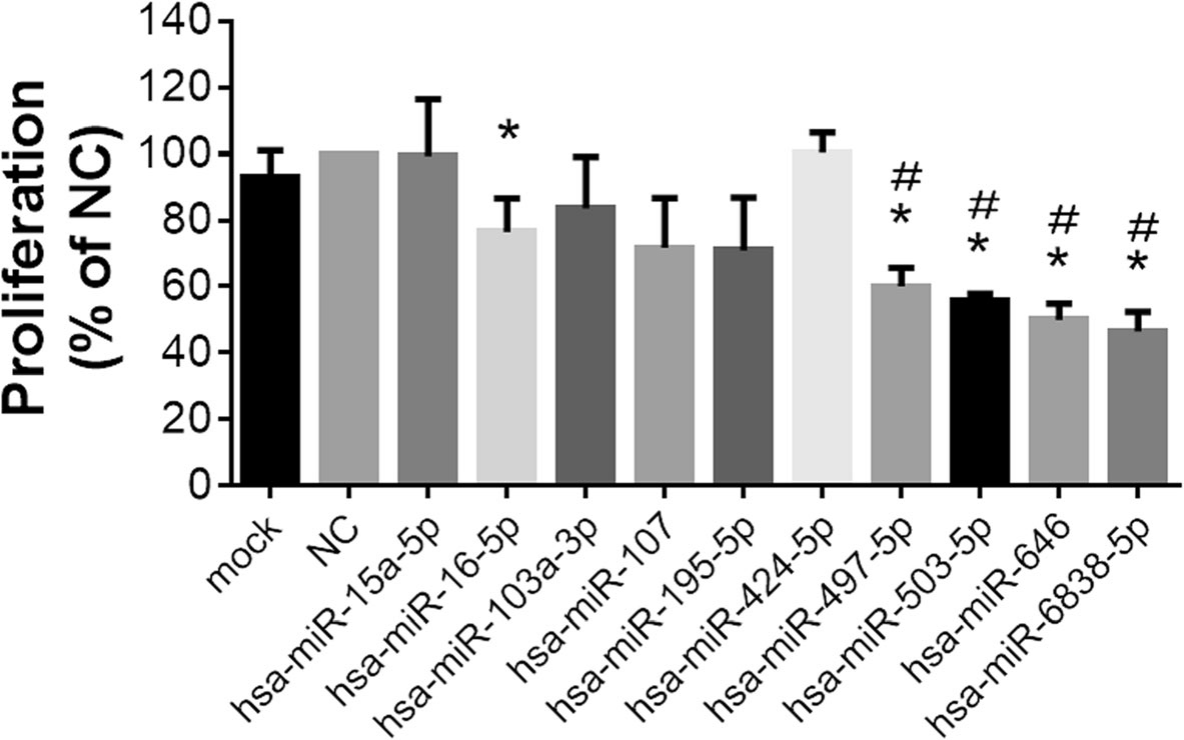
Cell proliferation during gain of miR-15/107 function in SW982 cells. Cell proliferation was detected by using CCK-8 assay after the SW982 cells were transfected with 10 nM miRNA mimic of the miR-15/107 family members for 48 h. Bar: mean ± SEM from 3 independent cell experiments, and 4 cell replicates were used in each cell experiment. *: *p* < 0.05 vs. NC (scramble miRNA mimic as negative control), #: *p* < 0.05 vs. mock (vehicle control)

Based on our previous signaling pathway prediction, this family may play an important role in the cell cycle pathway. We validated their role during cell proliferation in human synovial fibroblast cell line SW982 after gain of miR-15/107 function for 48 h. The results showed that the miRNA mimic for miR-16, miR-497, miR-503, miR-646 and miR-6838 displayed a significant inhibition on cell proliferation (Fig. 6). The results demonstrated that this gene family indeed has impact on cell cycle signaling as we predicted.

### Dysregulation of miR-15/107 in diseases and prospect of therapeutics

According to the archives in miR2Disease, dysregulated miR-15/107 family was reported in various human diseases, especially in cardiac hypertrophy [24], chronic lymphocytic leukemia (CLL) [25] and prostate cancer [26] (Table 3). More than 5 of such members were simultaneously found abnormally expressed in those diseases, while there are also some of the members individually participated in various diseases such as miR-103 in cerebellar neurodegeneration [27].

**Table 3:** Dysregulated miR-15/107 family in human diseases

	miR-15a	miR-15b	miR-16	miR-103	miR-107	miR-195	miR-424	miR-497	miR-503	sum
Adrenocortical carcinoma	-	-	-	-	-	-	-	-	√	1
Alzheimer's disease	√	-	-	-	√	-	-	-	-	2
Acute lymphoblastic leukemia (ALL)	-	-	-	-	-	-	√	-	-	1
Acute myeloid leukemia (AML)	-	√	-	√	-	√	√	-	-	4
Acute promyelocytic leukemia (APL)	√	√	-	-	-	-	-	-	-	2
Autism spectrum disorder (ASD)	√	√	-	-	-	-	-	-	-	2
B-cell chronic lymphocytic leukemia	-	√	-	-	-	-	-	-	-	1
Bladder cancer	-	-	-	-	-	√	-	-	-	1
Breast cancer	-	-	-	-	-	√	-	√	-	2
**Cardiac hypertrophy**	-	√	-	√	√	√	√	-	-	5
Cerebellar neurodegeneration	-	-	-	√	-	-	-	-	-	1
**Chronic lymphocytic leukemia (CLL)**	√	-	√	-	√	√	√	-	-	5
Chronic pancreatitis	-	-	-	-	-	√	-	√	-	2
Colorectal cancer	-	√	-	-	√	√	-	√	-	4
Endometriosis	-	-	-	-	-	-	√	-	-	1
Epithelial ovarian cancer (EOC)	-	-	-	√	-	-	-	-	-	1
Esophageal cancer	-	-	-	√	√	-	-	-	-	2
Gastric cancer (stomach cancer)	-	√	√	√	√	-	-	-	-	4
Glioma	√	√	√	-	-	-	-	-	-	3
Heart failure	-	-	-	-	-	√	-	-	-	1
Head and neck squamous cell carcinoma (HNSCC)	√	-	-	-	-	√	√	-	-	3
Hepatocellular carcinoma (HCC)	√	-	√	-	√	√	-	-	-	4
Hodgkin's lymphoma	-	-	√	-	-	-	-	-	-	1
Intrahepatic cholangiocarcinoma (ICC)	-	-	-	-	-	-	√	-	-	1
Kidney cancer	√	-	-	-	-	-	√	-	-	2
Lung cancer	-	-	√	-	-	√	-	√	-	3
Lupus nephritis	-	√	-	-	-	√	-	-	-	2
Malignant melanoma	√	-	-	-	√	-	-	-	-	2
Non-alcoholic fatty liver disease (NAFLD)	-	-	-	√	√	-	-	-	-	2
Non-small cell lung cancer (NSCLC)	√	√	√	-	√	-	-	-	-	4
Ovarian cancer (OC)	√	-	√	-	-	√	√	-	-	4
Oral Squamous Cell Carcinoma (OSCC)	-	-	√	-	√	-	-	-	-	2
Pancreatic cancer	-	√	-	√	√	-	√	-	-	4
Papillary thyroid carcinoma (PTC)	√	-	√	-	-	-	-	-	-	2
Pituitary adenoma	√	-	-	√	-	-	-	-	-	2
Polycystic Kidney Disease	√	-	-	-	-	-	-	-	-	1
Polycystic liver disease	√	-	-	-	-	-	-	-	-	1
**Prostate cancer**	√	-	√	√	-	√	-	√	√	6
Retinoblastoma	-	-	-	-	-	-	-	-	√	1
Schizophrenia	√	√	-	-	√	√	-	-	-	4
Serous ovarian cancer	-	-	√	-	-	-	-	-	-	1
Ulcerative colitis (UC)	-	-	√	-	-	√	-	-	-	2
Total (42)	17	12	13	10	13	16	10	5	3	

Diseases with more than 5 dysregulated miR-15/107 members were set in bold

These dysregulated miRNAs offered a prospect of therapeutics. Some lncRNAs were discovered to serve as miRNA sponge and since might alter disease progression. For example, miR-107 is found upregulated in glioma cell lines and binds to LncRNA nuclear paraspeckle assembly transcript 1 (NEAT1). NEAT1 silencing inhibits glioma progression, and NEAT1 induces glioma progression by regulating miR-107 as its endogenous sponge [28]. Besides, lncRNA RP11-79H23.3 might suppress the pathogenesis and development of bladder cancer by acting as a sponge for miR-107 to increase PTEN expression [29].

## Discussion

In this study, function and target interactions of miR-15/107 family was analyzed using various bioinformatics tools. Firstly, the evolutionary conservative “AGCAGC” sequence, possible clustered transcriptional pattern and tissue specific expression profile were described. Secondly, both the experimentally validated and bioinformatically predicted miRNA-target gene relationship of the entire family was fully interpreted to understand the mechanism of underlying collective effects. Moreover, pathway analysis among miR-15/107 family was performed and displayed in detail. Eventually, the dysregulation of miR-15/107 in diseases was discussed.

According to the previous reports, the implication of miR-15/107 family in various diseases has aroused much attention from multiple fields. In chronic lymphocytic lymphoma (CLL), downregulated miR-15a/miR-16-1 was widely studied [30–32]. Their CLL related target genes includes BCL2/MCL1/CCND1/WNT3A [30], BAZ2A/RNF41/RASSF5/MKK3/LRIG1 [31] et al. The miR-15/107 was also strongly implicated in Alzheimer’s disease. Alzheimer is firstly found to be associated with miR-107, it’s levels decreased significantly even in patients with the earliest stages of pathology [33]. The role of miR-15/107 family during AD pathogenesis was also found related with the target suppressing on CDK5R1/p35. Our study focuses on explaining how this miR-15/107 family displayed a collective function and similar implication in various human diseases. We agree that the major contributor should be the massive overlapping miRNA: mRNA target interaction shared crossing the entire family. For this part, we have displayed plenty of convincing evidence achieved from both the computationally prediction and the previous experimentally validation.

As above-mentioned, the common “AGCAGC” sequence within the 5′ seed binding region is the crucial characteristic of miR-15/107 family. Hence, plenty of shared target genes could be widely expected. Besides the important 5′ portion similarity, 3’portion also contribute to target binding. For example, 3′ portion of miR-103/107 appears to play a role in causing miRNAs to bind preferentially with CDS of target mRNAs [4].

We believe that there were also some additional clues from the transcriptional level of miRNA themselves. As shown in Table 2, there were several miRNA members within the miR-15/107 family transcribed in the same clusters including miR-15a-5p/miR-16-5p, miR-15b-5p/miR-16-5p, miR-195-5p/miR-497-5p, and miR-424-5p/miR-503-5p. It was considered that miRNAs in the same clusters might evolve to coordinately regulate the functionally related genes [34]. It is known that some evolutionarily conserved miRNAs are significantly enriched in miRNA clusters. According to this theory, the survival of new miRNAs in clusters is related to the function of pre-existing miRNAs in this cluster. For example, clustering miRNAs can synergistically target overlapping genomes, and new miRNAs can share this targeting function with the help of functional co-adaptation patterns, thereby successfully survive in clusters and performing other functions. This functional co-adaptation may be the driving force of clustering and persist in the initial stages of new miRNA clusters formation. Once their coordinated regulation of target genes is established, miRNA clusters will maintained by natural selection. Our existing data support this theory. We displayed that PANK is the host gene for miR-103/107, and this gene is known as a core player in the regulation of intracellular CoA. MiR-103/107 acts on genes in metabolic pathways in a synergistic manner with its host gene [35, 36]. Moreover, we considered that the coordinately regulating network derived from those clustered miRNAs might contribute to the majority of the present complicated regulation pattern to lots of target genes, and eventually lead to the multi-miRNA/target interaction network as we described.

## Conclusions

Our study proposes that the collective functions and implication of miR-15/107 family in various human diseases are achieved relying on the massive overlapping target genes. While the minor differences within target gene interaction among family members could also explain the signature behavior for some of the individual miRNA in aspects such as its disease-specific dysregulation and participation in pathways.

## Additional files

Additional file 1: Computationally predicted target interaction of miR-15/107 family (Targetscan database). (XLSX 578 kb)

Additional file 2: Experimentally validated target interaction of miR-15/107 family (TarBase v8.0). (XLSX 98 kb)

Additional file 3: Significantly involved KEGG pathways regulated by miR-15/107 family. (PDF 52 kb)

Additional file 4: List of miR-15/107 targeted genes within cell cycle pathway. (PDF 58 kb)

Additional file 5: Previous literatures showing the implication of miR-15/107 family and cell cycle related biological events. (PDF 130 kb)
