# Supplementary material for: Interpreting the MicroRNA-15/107 family: interaction identification by combining network based and experiment supported approach
Source: BMC Med Genet. 2019 May 31;20:96. doi: 10.1186/s12881-019-0824-9 (PMC6544937; doi:10.1186/s12881-019-0824-9)
Supplement: Supplementary file 3 — Significantly involved KEGG pathways regulated by miR-15/107 family. (PDF 52 kb) [file 12881_2019_824_MOESM3_ESM.pdf]

Additional file 3. Significantly involved KEGG pathways regulated by miR-15/107 family.

KEGG pathway	p-value	Involved target genes	Involved miRNAs
Fatty acid biosynthesis	<1E-325	5	6
Viral carcinogenesis	<1E-325	89	6
Fatty acid metabolism	<1E-325	21	7
Adherens junction	<1E-325	41	8
Proteoglycans in cancer	<1E-325	96	8
Hippo signaling pathway	1.11E-16	53	6
Hepatitis B	1.11E-16	67	8
p53 signaling pathway	5.61E-12	39	8
Fatty acid degradation	7.99E-12	15	5
TGF-beta signaling pathway	2.12E-11	33	6
Lysine degradation	2.39E-11	25	7
Cell cycle	2.51E-11	65	7
Protein processing in endoplasmic reticulum	5.77E-11	83	8
Prostate cancer	2.32E-10	49	8
Oocyte meiosis	2.04E-09	41	4
Glioma	2.84E-09	34	5
Chronic myeloid leukemia	9.47E-09	39	6
Fatty acid elongation	1.37E-07	8	5
Pathways in cancer	2.00E-07	141	5
Pancreatic cancer	3.15E-06	37	5
Colorectal cancer	7.36E-06	33	5
Melanoma	1.27E-05	26	4
Prion diseases	1.94E-05	10	1
Epstein-Barr virus infection	2.97E-05	72	5
Signaling pathways regulating pluripotency of stem cells	0.000109542	52	3
Progesterone-mediated oocyte maturation	0.000180975	33	4
Bladder cancer	0.000334588	24	6
Renal cell carcinoma	0.000551682	30	2
Small cell lung cancer	0.000558223	42	4
Non-small cell lung cancer	0.001344407	24	4
Glycosaminoglycan biosynthesis - chondroitin sulfate / dermatan sulfate	0.001670125	7	4
FoxO signaling pathway	0.001704236	37	2
Bacterial invasion of epithelial cells	0.002760089	25	2
Ubiquitin mediated proteolysis	0.002970495	45	3
Endometrial cancer	0.006452572	22	3
Shigellosis	0.01774476	26	2
Thyroid hormone signaling pathway	0.02261482	30	2
Dorso-ventral axis formation	0.02635076	16	3
HTLV-I infection	0.03037772	44	1
