# Supplementary material for: Interpreting the MicroRNA-15/107 family: interaction identification by combining network based and experiment supported approach
Source: BMC Med Genet. 2019 May 31;20:96. doi: 10.1186/s12881-019-0824-9 (PMC6544937; doi:10.1186/s12881-019-0824-9)
Supplement: Supplementary file 4 — List of miR-15/107 targeted genes within cell cycle pathway. (PDF 58 kb) [file 12881_2019_824_MOESM4_ESM.pdf]

Additional file 4. List of miR-15/107 targeted genes within cell cycle pathway

Gene symbol	Full name	Target miRNA	Validation methods
CCNA2	cyclin A2	miR-15a-5p, miR-15b-5p, miR-103a-3p, miR-107, miR-195-5p	HITS-CLIP
CCNB1	cyclin B1	miR-103a-3p, miR-107	HITS-CLIP
CCND3	cyclin D3	miR-15a-5p, miR-15b-5p, miR-16-5p, miR-195-5p	Multiple
CCNE1	cyclin E1	miR-15a-5p, miR-15b-5p, miR-16-5p, miR-103a-3p, miR-107, miR-195-5p, miR-424-5p	Multiple
CDC7	cell division cycle 7	miR-15a-5p, miR-15b-5p, miR-16-5p	Multiple
		miR-195-5p	HITS-CLIP
CDC14B	cell division cycle 14B	miR-15a-5p	HITS-CLIP
CDC20	cell division cycle 20	miR-16-5p	Multiple
CDC25A	cell division cycle 25A	miR-15a-5p, miR-15b-5p, miR-16-5p, miR-103a-3p, miR-107, miR-195-5p, miR-424-5p	Multiple
CDC25B	cell division cycle 25B	miR-15a-5p	HITS-CLIP
		miR-424-5p	Multiple
CDK1	cyclin dependent kinase 1	miR-103a-3p, miR-107	PAR-CLIP
CDK6	cyclin dependent kinase 6	miR-15a-5p, miR-15b-5p, miR-16-5p, miR-103a-3p, miR-107, miR-195-5p, miR-424-5p	Multiple
CDKN1A	cyclin dependent kinase inhibitor 1A	miR-15a-5p, miR-15b-5p, miR-16-5p, miR-103a-3p, miR-107, miR-195-5p, miR-424-5p	Multiple
CDKN2A	cyclin dependent kinase inhibitor 2A	miR-16-5p	Multiple
TGFB1	transforming growth factor beta 1	miR-103a-3p, miR-107	HITS-CLIP
SMAD3	SMAD family member 3	miR-16-5p	HITS-CLIP
		miR-424-5p	Multiple
SMAD4	SMAD family member 4	miR-103a-3p, miR-107	HITS-CLIP
SMC3	structural maintenance of chromosomes 3	miR-15a-5p, miR-15b-5p, miR-16-5p, miR-195-5p, miR-424-5p	PAR-CLIP
RBL1	RB transcriptional corepressor like 1	miR-15a-5p	PAR-CLIP
		miR-15b-5p, miR-16-5p, miR-103a-3p, miR-107, miR-195-5p, miR-424-5p	Multiple
CREBBP	CREB binding protein	miR-15a-5p, miR-15b-5p, miR-195-5p	HITS-CLIP
		miR-16-5p, miR-103a-3p, miR-107	Multiple
STAG2	stromal antigen 2	miR-424-5p	Multiple
GSK3B	glycogen synthase kinase 3 beta	miR-103a-3p, miR-107	Multiple
		miR-424-5p	PAR-CLIP

E2F3	E2F transcription factor 3	miR-15a-5p, miR-15b-5p, miR-16-5p, miR-195-5p, miR-424-5p	Multiple
		miR-103a-3p, miR-107	HITS-CLIP
E2F5	E2F transcription factor 5	miR-15a-5p, miR-15b-5p, miR-16-5p	Multiple
TFDP1	transcription factor Dp-1	miR-16-5p	HITS-CLIP
MDM2	MDM2 proto-oncogene	miR-15a-5p, miR-16-5p, miR-424-5p	Multiple
		miR-15b-5p, miR-103a-3p, miR-107, miR-195-5p	HITS-CLIP
PRKDC	protein kinase, DNA-activated, catalytic subunit	miR-15a-5p, miR-15b-5p, miR-16-5p, miR-195-5p	Multiple
		miR-424-5p	HITS-CLIP
TTK	TTK protein kinase	miR-15a-5p, miR-15b-5p, miR-16-5p, miR-195-5p	PAR-CLIP
RBX1	ring-box 1	miR-15a-5p, miR-15b-5p, miR-16-5p	PAR-CLIP
SKP2	S-phase kinase associated protein 2	miR-16-5p	HITS-CLIP
MYC	MYC proto-oncogene	miR-16-5p	TRAP
		miR-103a-3p, miR-107, miR-195-5p	Multiple
		miR-424-5p	PAR-CLIP
ZBTB17	zinc finger and BTB domain containing 17	miR-103a-3p, miR-107	PAR-CLIP
TP53	tumor protein p53	miR-15a-5p, miR-16-5p, miR-103a-3p, miR-107	Multiple
CHEK1	checkpoint kinase 1	miR-15a-5p, miR-15b-5p, miR-16-5p, miR-424-5p	Multiple
ANAPC13	anaphase promoting complex subunit 13	miR-15a-5p, miR-15b-5p, miR-16-5p, miR-195-5p, miR-424-5p	Multiple
		miR-103a-3p, miR-107	PAR-CLIP
GADD45A	growth arrest and DNA damage inducible gamma	miR-103a-3p, miR-107	HITS-CLIP
BUB1	BUB1 mitotic checkpoint serine/threonine kinase	miR-103a-3p, miR-107	Multiple
BUB3	BUB3 mitotic checkpoint serine/threonine kinase	miR-15a-5p, miR-15b-5p, miR-16-5p, miR-424-5p	PAR-CLIP
PCNA	proliferating cell nuclear antigen	miR-424-5p	PAR-CLIP
YWHAQ	tyrosine 3-monooxygenase/tryptophan 5-monooxygenase activation protein theta	miR-15a-5p	PAR-CLIP
		miR-16-5p	HITS-CLIP
		miR-424-5p	Multiple
PLK1	polo like kinase 1	miR-16-5p, miR-103a-3p, miR-107	Multiple
		miR-424-5p	PAR-CLIP
ABL1	ABL proto-oncogene 1, non-receptor tyrosine kinase	miR-16-5p, miR-103a-3p, miR-107, miR-424-5p	Multiple
WEE1	WEE1 homolog	miR-15a-5p, miR-15b-5p, miR-16-5p, miR-103a-3p, miR-107, miR-195-5p,	Multiple

		miR-424-5p	
HDAC2	histone deacetylase 2	miR-16-5p	HITS-CLIP
ORC6	origin recognition complex subunit 6	miR-15a-5p	PAR-CLIP
		miR-103a-3p, miR-107	Multiple
MCM3	minichromosome maintenance complex component 3	miR-16-5p	HITS-CLIP
MCM4	minichromosome maintenance complex component 4	miR-103a-3p, miR-107	HITS-CLIP
MCM7	minichromosome maintenance complex component 7	miR-15a-5p	PAR-CLIP
		miR-15b-5p, miR-16-5p, miR-103a-3p, miR-107, miR-424-5p	Multiple

HITS-CLIP: high-throughput sequencing of RNA isolated by crosslinking immunoprecipitation

PAR-CLIP: photoactivatable ribonucleoside-enhanced crosslinking and immunoprecipitation
