# Supplementary material for: Interpreting the MicroRNA-15/107 family: interaction identification by combining network based and experiment supported approach
Source: BMC Med Genet. 2019 May 31;20:96. doi: 10.1186/s12881-019-0824-9 (PMC6544937; doi:10.1186/s12881-019-0824-9)
Supplement: Supplementary file 5 — Previous literatures showing the implication of miR-15/107 family and cell cycle related biological events. (PDF 130 kb) [file 12881_2019_824_MOESM5_ESM.pdf]

Additional file 5. Previous literatures showing the implication of miR-15/107 family and cell cycle related biological events

miRNAs	Role in cell cycle related events	References
miR-15a-5p	Human miR-15a-5p inhibits pituitary tumor cell proliferation, invasion and migration by targeting Sox5.	(1)
	miR-15a inhibits cell proliferation and epithelial to mesenchymal transition in pancreatic ductal adenocarcinoma by down-regulating Bmi-1 expression.	(2)
	Up-regulation of miR-15a-5p suppresses cell proliferation, migration and invasion of these ectopic stem cells by targeting the 3' untranslated region of VEGFA.	(3)
	Increased miR-15a suppresses the Cyclin E1, regulated CDK2 and promotes the G_1/S transition.	(4)
	Overexpression of VEGFA or inhibition of miR-15a-5p promotes cell proliferation in osteoarthritis chondrocytes.	(5)
	miR-15a-5p suppresses the proliferation, motility, invasiveness and angiogenic potential and increased apoptosis when combined with docetaxel in chemotherapy-resistant ovarian cancer.	(6)
	High levels of miR-15a-5p in the young poor ovarian response group represses granulosa cell proliferation by regulating the PI3K-AKT-mTOR signaling pathway and promoted apoptosis through BCL2 and BAD.	(7)
	miR-15a-5p suppresses endometrial cancer cell growth via Wnt/β-catenin signaling pathway by inhibiting WNT3A.	(8)
	Ectopic overexpression of miR-15a-5p suppresses cancer proliferation, induces cell cycle arrest in HepG2 or SNU-182 cells *in vitro*, and inhibits HCC tumor growth *in vivo*.	(9)
miR-16-5p	MicroRNA-16-5p overexpression suppresses proliferation and invasion as well as triggers apoptosis by targeting VEGFA in breast carcinoma.	(10)
	Acute myeloid leukemia (AML) is associated with heterogeneous clonal proliferation, while numerous microRNAs are involved in AML and associated with the expression level of VEGF, including miR-16-5p.	(11)
miR-103a-3p	Overexpression of miR-103a-3p inhibits the proliferation	(12)

	and osteogenic differentiation of hADSCs (human adipose tissue-derived stromal cells), downregulates protein and mRNA levels of predicted target of miR-103a-3p (CDK6 and DICER1).	
	Over-expression of circTCF25 could down-regulate miR-103a-3p and miR-107, increase CDK6 expression, and promote proliferation and migration *in vitro* and *in vivo*.	(13)
miR-107	MicroRNA-107 promotes proliferation of gastriccancer cells by targeting cyclin dependent kinase 8.	(14)
	miR-107 promotes hepatocellular carcinoma cell proliferation by targeting Axin2.	(15)
	MicroRNA-107 inhibits tumor growth and metastasis by targeting the BDNF-mediated PI3K/AKT pathway in human non-small lung cancer.	(16)
	Up-regulation of MicroRNA-107 induces proliferation in human gastric cancer cells by targeting the transcription factor FOXO1.	(17)
	Up-regulation of miR-107 decreases proliferation rates, colony-forming abilities, tumorigenicity and increases apoptosis rates by targeting DKK-1.	(18)
	Treatment with miR-107 significantly blockes cell proliferation, DNA replication, colony formation, and invasion in SCC25 and CAL27 cells by targeting protein kinase Cε.	(19)
	miR-107 directly targets MCL1 and activates ATR/Chk1 pathway to inhibit proliferation, migration and invasiveness of cervical cancer cells.	(20)
	miR-107 overexpression inhibits cell proliferation in human neuroblastoma and ratpituitary adenoma cells by targeting AIP.	(21)
	Decreased miR-107 could inhibit tumor growth in a nude mouse model of triple-negative breast cancer.	(22)
	Overexpression of miR-107 in glioma cells leads to inhibition of HBMVEC proliferation, migration and tube formation ability.	(23)
	MicroRNA-107 inhibits U87 glioma cell growth and invasion by target Notch2.	(24)
	miR-107 overexpression suppresses cell proliferation in	(25)

	glioma cells, whereas miR-107 knockdown promotes cell growth in MO59K cell line.	
	P53-induced microRNA-107 inhibits proliferation of glioma cells and down-regulates the expression of CDK6 and Notch-2.	(26)
	MiR-107 suppresses proliferation of hepatoma cells through targeting HMGA2 mRNA 3'UTR	(27)
	miR-107 can simultaneously target large tumor suppressor 2 (LATS2) and synchronically modulate its expression, thereby promoting proliferation and invasion of GAC cells.	(28)
	miR-107 improves the proliferative and migratory ability of HepG2 cells.	(29)
	miR-107 mimic leads to a significant increase in the G_0/G_1 population and a significant decrease in the G_2/M population, and functions as a tumor suppressor in human esophageal squamous cell carcinoma.	(30)
miR-195-5p	Overexpression of miR-195-5p significantly suppresses renal cell carcinoma growth in vitro and in vivo by targeting REGγ.	(31)
	Overexpression of miR-195-5p inhibits cell proliferation, reduces cell colony formation, suppresses cell migration and causes an accumulation of cells in the G_1 phase of the cell cycle by regulating CCNE1 in breast cancer.	(32)
	miR-195-5p suppresses the proliferation, migration, and invasion of oral squamous cell carcinoma by targeting TRIM14.	(33)
	Overexpression of miR-195-5p in hepatoma cells reduces PHF19 expression, leads to suppression of hepatoma cell invasion, migration and proliferation in *vitro*.	(34)
	MicroRNA-195-5p suppresses glucose uptake and proliferation of human bladder cancer T24 cells by regulating GLUT3 expression.	(35)
	MicroRNA-195-5p suppresses osteosarcoma cell proliferation and invasion by suppressing naked cuticle homolog 1.	(36)
	Anti-apoptosis endothelial cell-secreted microRNA-195-5p promotes pulmonary arterial smooth muscle cell proliferation and migration in pulmonary arterial hypertension.	(37)

miR-424-5p	miR-424-5p promotes proliferation of gastric cancer by targeting Smad3 through TGF-β signaling pathway.	(38)
	Downregulated miR-424-5p causes the overexpression of MEK1 and cyclin E1 in the psoriatic keratinocytes, resulting in the keratinocyte overgrowth and hyper-proliferation.	(39)
	miR-424-5p mimics could decrease the invasion, metastasis and proliferation of esophageal squamous carcinom cells, while SMAD7 is a specific target gene for miR-424-5p.	(40)
	Ectopic expression of miR-424 promotes proliferation and invasion of gastric cancer cells by targeting LATS1 gene.	(41)
	miR-424-5p plays its role as an anti-onco gene in cervical cancer cell growth by targeting KDM5B via the Notch signaling pathway.	(42)
	miR-424-5p increases proliferation, migration and invasion of pancreatic cancer cells, while inhibits cell apoptosis.	(43)
	miR-424-5p could prevent the proliferation, migration, and invasion in NSCLC cells.	(44)
miR-497-5p	miR-497-5p inhibits cell proliferation and invasion by targeting KCa3.1 in angiosarcoma.	(45)
miR-503-5p	miR-503-3p (the passenger strand of miR-503-5p) inhibits cell proliferation in lung cancer.	(46)
miR-646	miR-646 negatively regulates NOB1 and inhibits the proliferation and migration of renal cancer cells.	(47)
	miR-646 inhibits cell proliferation by targeting FOXK1 in gastric cancer.	(48)
	Overexpression of miR-646 inhibits cell proliferation and metastasis in lung cancer.	(49)
	Overexpression of miR-646 inhibits cell proliferation, migration, and invasion in osteosarcoma cells.	(50)
miR-6838-5p	None	-

References:

1. Renjie W, Haiqian L. MiR-132, miR-15a and miR-16 synergistically inhibit pituitary tumor cell proliferation, invasion and migration by targeting Sox5. Cancer letters. 2015;356(2):568-78.
2. Guo S, Xu X, Tang Y, Zhang C, Li J, Ouyang Y, et al. miR-15a inhibits cell proliferation and epithelial to mesenchymal transition in pancreatic ductal adenocarcinoma by down-regulating Bmi-1 expression. Cancer letters. 2014 Mar 1;344(1):40-6.
3. Liu XJ, Bai XG, Teng YL, Song L, Lu N, Yang RQ. miRNA-15a-5p regulates VEGFA in endometrial mesenchymal stem cells and contributes to the pathogenesis of endometriosis. European review for

medical and pharmacological sciences. 2016 Aug;20(16):3319-26.
4. Alderman C, Yang Y. The anti-melanoma activity and oncogenic targets of hsa-miR-15a-5p. RNA & disease. 2016;3(4).
5. Chen H, Tian Y. MiR-15a-5p regulates viability and matrix degradation of human osteoarthritis chondrocytes via targeting VEGFA. Bioscience trends. 2017 Jan 16;10(6):482-8.
6. Rodriguez-Aguayo C, Monroig PDC, Redis RS, Bayraktar E, Almeida MI, Ivan C, et al. Regulation of hnRNPA1 by microRNAs controls the miR-18a-K-RAS axis in chemotherapy-resistant ovarian cancer. Cell discovery. 2017;3:17029.
7. Zhang K, Zhong W, Li WP, Chen ZJ, Zhang C. miR-15a-5p levels correlate with poor ovarian response in human follicular fluid. Reproduction. 2017 Oct;154(4):483-96.
8. Wang ZM, Wan XH, Sang GY, Zhao JD, Zhu QY, Wang DM. miR-15a-5p suppresses endometrial cancer cell growth via Wnt/beta-catenin signaling pathway by inhibiting WNT3A. European review for medical and pharmacological sciences. 2017 Nov;21(21):4810-8.
9. Long J, Jiang C, Liu B, Fang S, Kuang M. MicroRNA-15a-5p suppresses cancer proliferation and division in human hepatocellular carcinoma by targeting BDNF. Tumour Biol. 2016 May;37(5):5821-8.
10. Qu Y, Liu H, Lv X, Liu Y, Wang X, Zhang M, et al. MicroRNA-16-5p overexpression suppresses proliferation and invasion as well as triggers apoptosis by targeting VEGFA expression in breast carcinoma. Oncotarget. 2017 Sep 22;8(42):72400-10.
11. Li L, Zhu L, Wang Y, Zhou, Zhu J, Xie W, et al. Profiling of microRNAs in AML cells following overexpression or silencing of the VEGF gene. Oncology letters. 2017 Jan;13(1):105-10.
12. Kim DS, Lee SY, Lee JH, Bae YC, Jung JS. MicroRNA-103a-3p controls proliferation and osteogenic differentiation of human adipose tissue-derived stromal cells. Experimental & molecular medicine. 2015 Jul 10;47:e172.
13. Zhong Z, Lv M, Chen J. Screening differential circular RNA expression profiles reveals the regulatory role of circTCF25-miR-103a-3p/miR-107-CDK6 pathway in bladder carcinoma. Scientific reports. 2016 Aug 3;6:30919.
14. Song YQ, Ma XH, Ma GL, Lin B, Liu C, Deng QJ, et al. MicroRNA-107 promotes proliferation of gastric cancer cells by targeting cyclin dependent kinase 8. Diagnostic pathology. 2014 Aug 28;9:164.
15. Zhang JJ, Wang CY, Hua L, Yao KH, Chen JT, Hu JH. miR-107 promotes hepatocellular carcinoma cell proliferation by targeting Axin2. International journal of clinical and experimental pathology. 2015;8(5):5168-74.
16. Xia H, Li Y, Lv X. MicroRNA-107 inhibits tumor growth and metastasis by targeting the BDNF-mediated PI3K/AKT pathway in human non-small lung cancer. International journal of oncology. 2016 Oct;49(4):1325-33.
17. Li F, Liu B, Gao Y, Liu Y, Xu Y, Tong W, et al. Upregulation of microRNA-107 induces proliferation in human gastric cancer cells by targeting the transcription factor FOXO1. FEBS letters. 2014 Feb 14;588(4):538-44.
18. Zhang ZC, Liu JX, Shao ZW, Pu FF, Wang BC, Wu Q, et al. In vitro effect of microRNA-107 targeting Dkk-1 by regulation of Wnt/beta-catenin signaling pathway in osteosarcoma. Medicine. 2017 Jul;96(27):e7245.
19. Datta J, Smith A, Lang JC, Islam M, Dutt D, Teknos TN, et al. microRNA-107 functions as a candidate tumor-suppressor gene in head and neck squamous cell carcinoma by downregulation of protein kinase Cvarepsilon. Oncogene. 2012 Sep 6;31(36):4045-53.
20. Zhou C, Li G, Zhou J, Han N, Liu Z, Yin J. miR-107 activates ATR/Chk1 pathway and suppress

cervical cancer invasion by targeting MCL1. PloS one. 2014;9(11):e111860.
21. Trivellin G, Butz H, Delhove J, Igreja S, Chahal HS, Zivkovic V, et al. MicroRNA miR-107 is overexpressed in pituitary adenomas and inhibits the expression of aryl hydrocarbon receptor-interacting protein in vitro. American journal of physiology Endocrinology and metabolism. 2012 Sep 15;303(6):E708-19.
22. Zhang L, Ma P, Sun LM, Han YC, Li BL, Mi XY, et al. MiR-107 down-regulates SIAH1 expression in human breast cancer cells and silencing of miR-107 inhibits tumor growth in a nude mouse model of triple-negative breast cancer. Mol Carcinog. 2016 May;55(5):768-77.
23. Chen L, Li ZY, Xu SY, Zhang XJ, Zhang Y, Luo K, et al. Upregulation of miR-107 Inhibits Glioma Angiogenesis and VEGF Expression. Cell Mol Neurobiol. 2016 Jan;36(1):113-20.
24. Chen L, Chen XR, Chen FF, Liu Y, Li P, Zhang R, et al. MicroRNA-107 inhibits U87 glioma stem cells growth and invasion. Cell Mol Neurobiol. 2013 Jul;33(5):651-7.
25. He J, Zhang W, Zhou Q, Zhao T, Song Y, Chai L, et al. Low-expression of microRNA-107 inhibits cell apoptosis in glioma by upregulation of SALL4. The international journal of biochemistry & cell biology. 2013 Sep;45(9):1962-73.
26. Chen L, Zhang R, Li P, Liu Y, Qin K, Fa ZQ, et al. P53-induced microRNA-107 inhibits proliferation of glioma cells and down-regulates the expression of CDK6 and Notch-2. Neurosci Lett. 2013 Feb 8;534:327-32.
27. Wang Y, Chen F, Zhao M, Yang Z, Zhang S, Ye L, et al. MiR-107 suppresses proliferation of hepatoma cells through targeting HMGA2 mRNA 3'UTR. Biochemical and biophysical research communications. 2016 Nov 18;480(3):455-60.
28. Zhang M, Wang X, Li W, Cui Y. miR-107 and miR-25 simultaneously target LATS2 and regulate proliferation and invasion of gastric adenocarcinoma (GAC) cells. Biochemical and biophysical research communications. 2015 May 8;460(3):806-12.
29. Zou C, Zou C, Cheng W, Li Q, Han Z, Wang X, et al. Heme oxygenase-1 retards hepatocellular carcinoma progression through the microRNA pathway. Oncology reports. 2016 Nov;36(5):2715-22.
30. Sharma P, Saini N, Sharma R. miR-107 functions as a tumor suppressor in human esophageal squamous cell carcinoma and targets Cdc42. Oncology reports. 2017 May;37(5):3116-27.
31. Chen S, Wang L, Yao X, Chen H, Xu C, Tong L, et al. miR-195-5p is critical in REGgamma-mediated regulation of wnt/beta-catenin pathway in renal cell carcinoma. Oncotarget. 2017 Sep 8;8(38):63986-4000.
32. Luo Q, Wei C, Li X, Li J, Chen L, Huang Y, et al. MicroRNA-195-5p is a potential diagnostic and therapeutic target for breast cancer. Oncology reports. 2014 Mar;31(3):1096-102.
33. Wang T, Ren Y, Liu R, Ma J, Shi Y, Zhang L, et al. miR-195-5p Suppresses the Proliferation, Migration, and Invasion of Oral Squamous Cell Carcinoma by Targeting TRIM14. BioMed research international. 2017;2017:7378148.
34. Xu H, Hu YW, Zhao JY, Hu XM, Li SF, Wang YC, et al. MicroRNA-195-5p acts as an anti-oncogene by targeting PHF19 in hepatocellular carcinoma. Oncology reports. 2015 Jul;34(1):175-82.
35. Fei X, Qi M, Wu B, Song Y, Wang Y, Li T. MicroRNA-195-5p suppresses glucose uptake and proliferation of human bladder cancer T24 cells by regulating GLUT3 expression. FEBS letters. 2012 Feb 17;586(4):392-7.
36. Qu Q, Chu X, Wang P. MicroRNA-195-5p suppresses osteosarcoma cell proliferation and invasion by suppressing naked cuticle homolog 1. Cell Biol Int. 2017 Mar;41(3):287-95.
37. Zeng Z, Yao J, Li Y, Xue Y, Zou Y, Shu Z, et al. Anti-apoptosis endothelial cell-secreted

microRNA-195-5p promotes pulmonary arterial smooth muscle cell proliferation and migration in pulmonary arterial hypertension. Journal of cellular biochemistry. 2017 Sep 1.

38. Wei S, Li Q, Li Z, Wang L, Zhang L, Xu Z. miR-424-5p promotes proliferation of gastric cancer by targeting Smad3 through TGF-beta signaling pathway. Oncotarget. 2016 Nov 15;7(46):75185-96.

39. Jinnin M. Recent progress in studies of miRNA and skin diseases. The Journal of dermatology. 2015 Jun;42(6):551-8.

40. Wang F, Wang J, Yang X, Chen D, Wang L. MiR-424-5p participates in esophageal squamous cell carcinoma invasion and metastasis via SMAD7 pathway mediated EMT. Diagnostic pathology. 2016 Sep 15;11(1):88.

41. Zhang J, Liu H, Hou L, Wang G, Zhang R, Huang Y, et al. Circular RNA_LARP4 inhibits cell proliferation and invasion of gastric cancer by sponging miR-424-5p and regulating LATS1 expression. Molecular cancer. 2017 Sep 11;16(1):151.

42. Zhou Y, An Q, Guo RX, Qiao YH, Li LX, Zhang XY, et al. miR424-5p functions as an anti-oncogene in cervical cancer cell growth by targeting KDM5B via the Notch signaling pathway. Life Sci. 2017 Feb 15;171:9-15.

43. Wu K, Hu G, He X, Zhou P, Li J, He B, et al. MicroRNA-424-5p suppresses the expression of SOCS6 in pancreatic cancer. Pathol Oncol Res. 2013 Oct;19(4):739-48.

44. Zhang M, Zeng J, Zhao Z, Liu Z. Loss of MiR-424-3p, not miR-424-5p, confers chemoresistance through targeting YAP1 in non-small cell lung cancer. Mol Carcinog. 2017 Mar;56(3):821-32.

45. Chen Y, Kuang D, Zhao X, Chen D, Wang X, Yang Q, et al. miR-497-5p inhibits cell proliferation and invasion by targeting KCa3.1 in angiosarcoma. Oncotarget. 2016 Sep 6;7(36):58148-61.

46. Sun Y, Li L, Xing S, Pan Y, Shi Y, Zhang L, et al. miR-503-3p induces apoptosis of lung cancer cells by regulating p21 and CDK4 expression. Cancer Biomark. 2017 Dec 6;20(4):597-608.

47. Li W, Liu M, Feng Y, Xu YF, Huang YF, Che JP, et al. Downregulated miR-646 in clear cell renal carcinoma correlated with tumour metastasis by targeting the nin one binding protein (NOB1). British journal of cancer. 2014 Sep 9;111(6):1188-200.

48. Zhang P, Tang WM, Zhang H, Li YQ, Peng Y, Wang J, et al. MiR-646 inhibited cell proliferation and EMT-induced metastasis by targeting FOXK1 in gastric cancer. British journal of cancer. 2017 Aug 8;117(4):525-34.

49. Pan Y, Chen Y, Ma D, Ji Z, Cao F, Chen Z, et al. miR-646 is a key negative regulator of EGFR pathway in lung cancer. Experimental lung research. 2016 Aug;42(6):286-95.

50. Sun XH, Geng XL, Zhang J, Zhang C. miRNA-646 suppresses osteosarcoma cell metastasis by downregulating fibroblast growth factor 2 (FGF2). Tumour Biol. 2015 Mar;36(3):2127-34.
